# Plant Viral Metagenomic Analysis from a Preliminary Field Survey in Angola Reveals Complex Mixed Infections in Vegetable Crops

**DOI:** 10.3390/v18080822

**Published:** 2026-07-26

**Authors:** Serafina Serena Amoia, Annalisa Giampetruzzi, Fernando Francisco de Sousa Neto, Luisa Flora António, Adérito Tomás Pais da Cunha, Angelantonio Minafra

**Affiliations:** 1 Institute for Sustainable Plant Protection–CNR, 70126 Bari, Italy; serafinaserena.amoia@cnr.it (S.S.A.); annalisa.giampetruzzi@cnr.it (A.G.); 2 Instituto Superior Politécnico do Cuanza Sul, Sumbe P.O. Box 82, Angola; fernando.neto@ispcs.ao (F.F.d.S.N.); flora.antonio@ispcs.ao (L.F.A.); aderito.cunha@ciencia.ao (A.T.P.d.C.); 3 Centro Nacional de Investigação Cientifica, Luanda P.O. Box 34, Angola

**Keywords:** viral metagenomics, high-throughput sequencing (HTS), mixed infections, vector-borne plant viruses, plant health surveillance

## Abstract

Climatic changes are heavily affecting the sustainability of vegetable crops crucial for food supply worldwide, mainly in subtropical countries. One of the main threats to food security is the spread of diseases caused by plant viruses, favored by irregular rains and extreme temperatures, which reduce crop yield and quality. During a preliminary field survey carried out in two provinces of Angola in 2024, a few symptomatic plants of tomato, habanero pepper, common bean and a wild weed were sampled. These plants generally showed dwarfing, yellowing and leaf curl and were submitted to high-throughput sequencing to detect any viral agent. The evidence of mixed infections of several polyphagous viruses with RNA or DNA genomes, variously affecting the selected plants, was assessed from the sequence analysis and further confirmed for most samples by molecular tests, like (RT)-PCR or qPCR. Emerging polero-, begomo and tobamoviruses were denoted as infecting these plants. A novel, previously unknown carlavirus was also described in a wild weed. Most of those viruses are efficiently mechanically transmitted or airborne vehiculated by insect vectors. Although based on a limited number of samples, this study provides a first insight into the diversity of viruses infecting vegetable crops in Angola. It also highlights the pressing need for a broader monitoring to better understand virus distribution and epidemiology, and suggests the use of virus-free seeds to reduce the potential risk to crop production.

## 1. Introduction

The effects of climatic changes on agriculture and food production can be sometimes dramatic in the global south sub-tropical countries that are often severely exposed to sudden changes in climatic parameters (rain, temperature, hygrometry, etc.). To face these effects, irrigation and soil management practices must be carefully applied for specific conditions and crops [1]. Moreover, pests and diseases heavily hamper yield and quality either in the field or in post-harvest, and dangerous vector-borne pathogens can be easily spread without containment [2]. Several taxa of viruses that affect world-wide important vegetable crops include begomo-, tospo-, cucumo-, polero- and potyviruses, and are efficiently transmitted by airborne insect vectors [3]. Some other viruses, like emerging tobamoviruses, are easily mechanically transmitted or seed-borne [4].

Climatic conditions, like high temperature values in the dry season and irregular rain during crop growth, could help viruses and vectors proliferate. In Angola, May represents the start of the dry season in the central climatic regions, including the provinces of Cuanza Sul and Benguela. In the months from April to October, the rainfall rate is well under 100 mm *per* month, while the annual average is around 85.5 mm [5]. Notwithstanding the difficulties due to a mainly rain-fed agriculture, in Angola the increasing production of arable crops like legumes, in particular bean, and vegetables, mainly tomato, ranging over 2 million MT, had a consistent boost of 7.1% in the last documented campaign 2023–2024 compared to the previous one (source: Ministerio de Agricultura e Florestas, Instituto Nacional de Estatística de Angola 2024; https://www.ine.gov.ao/publicacoes/detalhes/NDY0MDE%3D. Accessed on 20 December 2025). In this production, the Provinces of Cuanza Sul and Benguela account for about 30% of the national yield.

A principal drawback in the low-income farmer economy is that the main source of vegetable seeds (more than 90%) is carried over by the yearly self-production. This uncontrolled batch of propagation, even if selected by visual inspection performed by farmers for disease symptoms, can certainly represent a risky supply for the spread of systemic and seed-borne pathogens. Another underestimated source of potential virus contamination for the crops is the spill-over that could occur when wild weeds at the plot interface are infected, sometimes latently, with viruses that can be transmitted and spread [6,7]. Considering this scenario, monitoring of the crops to detect early symptom expression, and its reinforcement through specific or even generic diagnostic tests [8], is of paramount importance for containment strategies against the spread of viral diseases. Indeed, an increasing number of reports have highlighted the presence of known and even unknown viruses at the agro-ecological interface [9,10] and the problems caused by their potential diffusion. Most of these studies benefited from the broad application of high-throughput sequencing (HTS) to nucleic acid extracts, allowing the identification of viruses and viroids affecting both cultivated crops and wild weeds within the same fields [11,12]. This methodology of investigation, mainly based on pooled samples, can represent a useful approach to reducing the cost of analysis and provides a wide snapshot of viruses’ presence at an epidemiological scale.

International cooperation can often be a valuable tool for fostering coordinated actions and shared partnerships among the different actors (farmers, technicians, researchers, policy decision makers) in less developed countries. In the frame of the EU-funded project RE-FARM (Research on agroecological innovations for increasing resilience to climate change in Cuanza Sul and Benguela; EuropeAid/171171/DD/ACT/Multi), a small survey for the presence of virus-derived symptoms was carried out in June 2024 around some experimental fields in the provinces of Benguela and Cuanza Sul (Angola). This report describes the complex array of viruses retrieved in the few sampled plants tested by HTS analysis and highlights the potential risk that such viruses could represent without an active containment strategy. 

## 2. Materials and Methods

**Figure 1:**
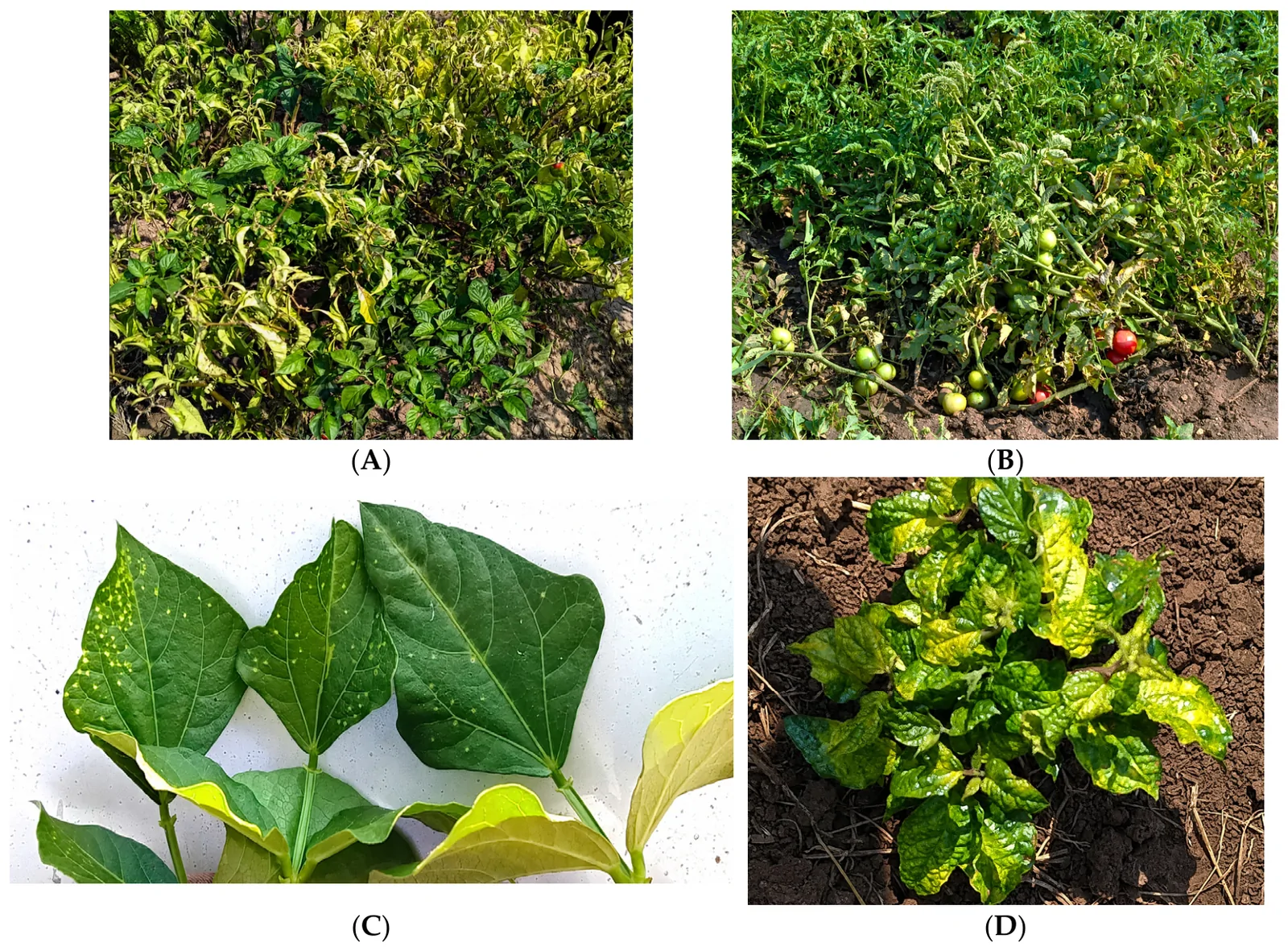
Virus-like symptoms observed during the survey on (**A**) pepper plants (yellowing and dwarfing); (**B**) tomato plants (stunting and leaf upward curling); (**C**) bean (small necrotic spots); and (**D**) a lamiaceous weed (yellow mosaic).

During a survey in June 2024 in two different fields in Angola, our attention was focused on the occurrence of virus-derived symptoms on crops and weeds. The first inspected plot was the habanero pepper (*Capsicum chinense*, Jacq.) cultivated in an irrigated field, consociated with banana and maize, close to Dombe Grande, province of Benguela. Plants were already in the harvest period but showed yellowing, dwarfing by shortening of internodes, smaller size of leaves with upward curling, and unripe fruits (Figure 1A). The second plot visited was in Sungo do Galo (Municipality of Seles, Province of Cuanza Sul). Here, close to an intercropped maize and bean plot, not irrigated and managed by the RE-FARM project, three different plants were selected: (i) tomato (*Solanum lycopersicum*, L.) plants, from a plot spanning a couple of hectares, with strong curling and yellowing symptoms; (ii) bean (*Phaseolus vulgaris,* L.) plants showing small decolorated spots, which tended to degenerate to necrotic ones in the older leaves, (Figure 1B,C); and (iii) a wild weed, most likely belonging to the Lamiaceae family, exhibiting patches of a striking yellow-chrome mosaic on the leaves [13] (Figure 1D).

Total RNA fraction was extracted from leaf tissues of a single plant *per* plot that was representative of the featured symptoms, using the Cetyltrimethylammonium Bromide (CTAB) method followed by a LiCl precipitation [14]. Optical density measurements and gel electrophoresis of the extracted RNAs were evaluated before sending for HTS analysis, using the rRNA-depleted total RNA protocol, to an external service (Macrogen, Seul, South Korea). Four libraries were prepared and named accordingly: AngH for habanero pepper, AngD for the wild weed, AngF for bean, and AngT for tomato. The sequencing was performed on an Illumina NextSeq2000 platform (2 × 150 bp), obtaining an average of 50 million raw reads (see Supplementary Table S1 for detailed information about each library). The quality of the libraries was assessed by the FastQC tool (https://www.bioinformatics.babraham.ac.uk/projects/fastqc/; accessed on 10 December 2025), and the adapter trimming was performed using the BBDuk tool implemented on the Geneious Prime^®^ platform version 2025.0.3. De novo assembly was done by the SPAdes software v3.11.1 [15], either on Geneious Prime^®^ or Galaxy platform, retrieving an average of 117,851 global contigs for the four libraries. Preliminarily, the contigs were annotated using BLASTN against a nucleotide plant virus database (custom-selected from https://www.ncbi.nlm.nih.gov/nucleotide/; accessed on 15 December 2025) for the identification of known viruses. Subsequently, BLASTX searches were performed against a custom database of plant viral proteins to identify distant similarities with known viruses, thereby facilitating the detection and characterization of potentially novel viral species. In this latter analysis, the thresholds were set at an e-value of <10^−4^ and a percent identity higher than 50% on an alignment length of at least 50 amino acids. At the end of the process, several contigs of variable lengths (from a few hundred (ca. 300) to thousands of (8500) nucleotides) matching plant virus sequences were positively selected (Table 1).

To further assess the consistency of the identified viruses, paired-end reads of each library were mapped on the reference genomes derived from the major BLASTX hits using Bowtie2 v. 2.3.5 [16] and the output visualized through Geneious Prime.

Based on the consensus of several identified virus sequences, primer sets were designed for PCR amplification. Primer pair selection was done either manually on the pairwise alignments obtained by BLASTN with the closest hits, through multiple alignments with reference genomes, or employing Primer 3 software (https://primer3.ut.ee/; accessed on 20 January 2026) on single contigs (Supplementary Table S2). Random hexamer-primed cDNA was synthesized on 500 ng of total RNA by M-MLV RTase (Thermo Fisher Scientific, Waltham, MA, USA) and used as template for conventional or quantitative PCR. The PCR reaction mix was set up as follows: 2× GoTaq^®^ Green Master Mix (Promega Corporation, Madison, WI, USA), 200 nM of each primer and 1 μL of cDNA were added to nuclease-free H_2_O, resulting in a final volume of 20 μL. The PCR program included one cycle at 95 °C for 3 min, 35 cycles at 94 °C for 30 s, 58 °C for 30 s, and 72 °C for 30 s, followed by a final extension at 72 °C for 10 min. qPCR was carried out on a CFX96 real-time thermocycler using a 2× Fast SYBR™ Green Master Mix (Applied Biosystems, Foster City, CA, USA) for 40 cycles of 95 °C/15 s and 65 °C/45 s, followed by melting curve analysis. All the amplicons obtained in conventional PCR were purified and sequenced by the Sanger method (Macrogen, Amsterdam, The Netherlands) in both directions with the corresponding amplification primers.

## 3. Results

The reduced batch of symptomatic plants that was possible to sample and process in the survey was not apparently a limiting factor for the identification of several viral agents. Indeed, the sequenced libraries returned a picture of a quite variable presence of mixed infections of viruses belonging to different taxonomical families and genome kinds (RNA or DNA). The low representation of viroids in the sequenced libraries could be due either to the low titer of these agents in the tissues or to a bias in the extraction method that does not enable for the enrichment of lower molecular weight RNA fractions.

Generally, after the characterization by the bioinformatic pipelines, at least two viruses *per* library were retrieved and confirmed by specific RT/q-PCR, followed by amplicon sequencing or melting curve analysis. In this section, the most relevant information gained from the analysis of the four libraries is summarized, focusing on viruses featuring a high number of mapped reads, a conspicuous average length of contigs and statistical support as BLASTX e-value (Table 1; Supplementary Table S3).

### 3.1. Viruses in a Wild Weed (AngD_Library)

A few of these plants were spontaneously growing close to the cultivated plot and were highly visible for their marked lively yellow-green colors. It was not possible to unequivocally classify the plant into a specific family or genus, since no other botanical features were visible except for the leaves. However, from the molecular point of view, several plant-derived transcripts analysed as BLASTN/X returned significant hits associated with members of the Lamiaceae family (data not shown).

**Figure 2:**
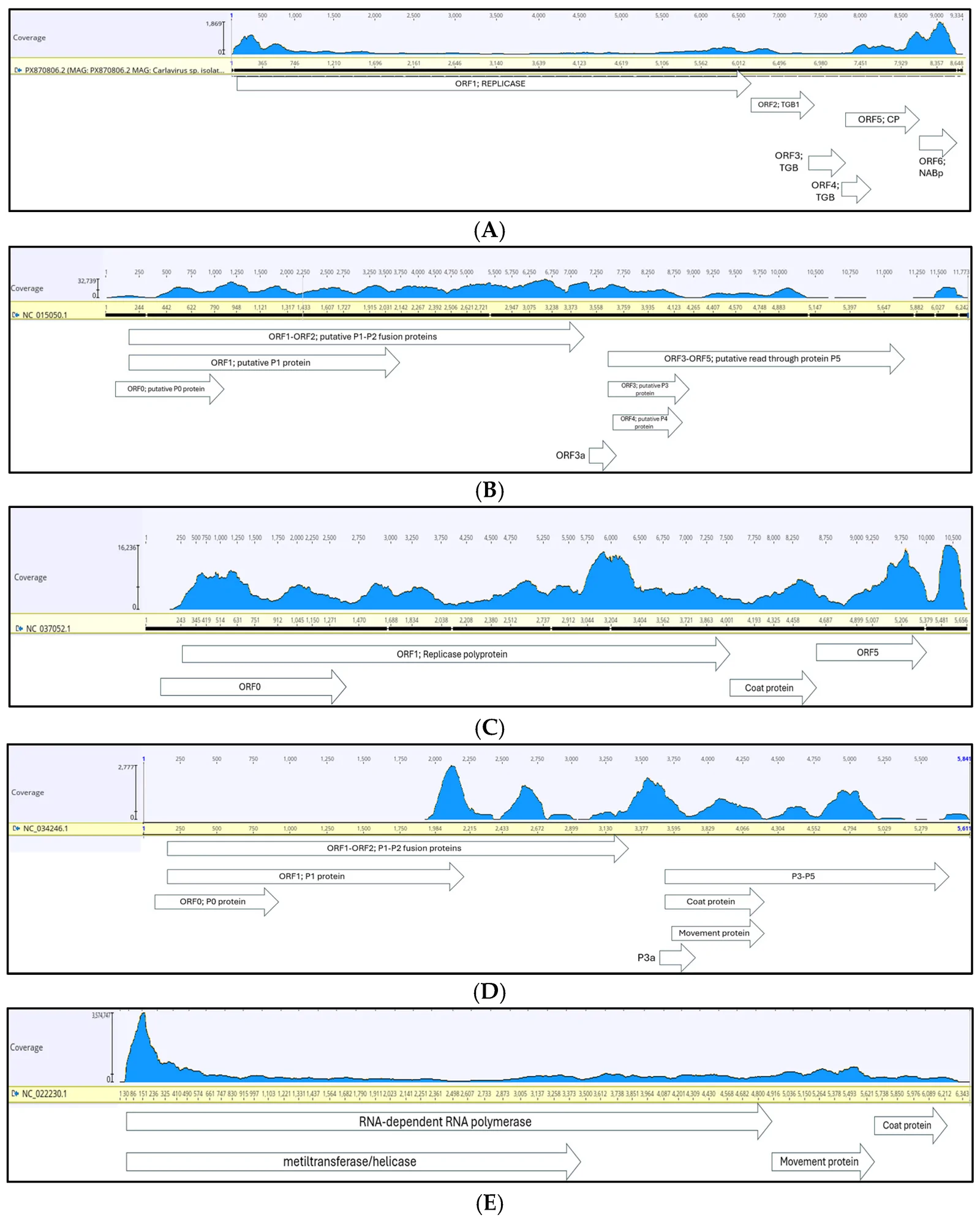

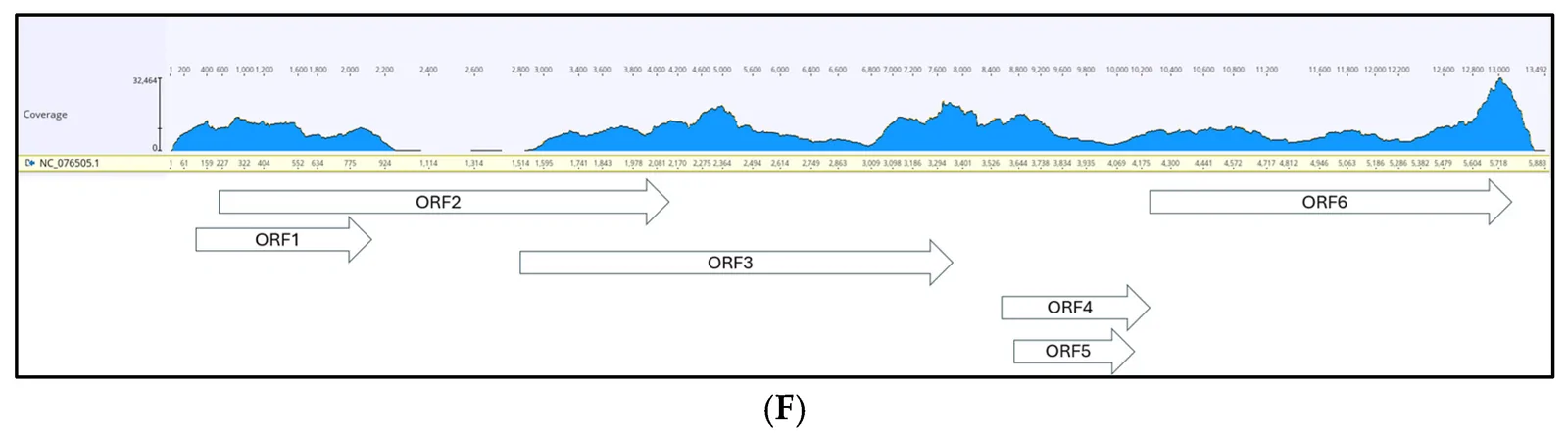
Coverage maps of HTS reads aligned to the complete reference genomes of (**A**) Seles weed carlavirus; (**B**) Pepper vein yellows virus; (**C**) Pepper enamovirus; (**D**) Cowpea polerovirus 1; (**E**) Tomato mottle mosaic virus, and (**F**) Potato leafroll virus. The blue histograms represent the coverage profile along the reference viral genomes, whereas arrows indicate the positions of the annotated genes and open reading frames (ORFs). Accession numbers of the reference genomes are indicated in the yellow banners and genome expression is reported by ORF arrows in the lower panel. CP: coat protein; TGB: triple gene block; NABp: nucleic acid binding protein.

**Figure 3:**
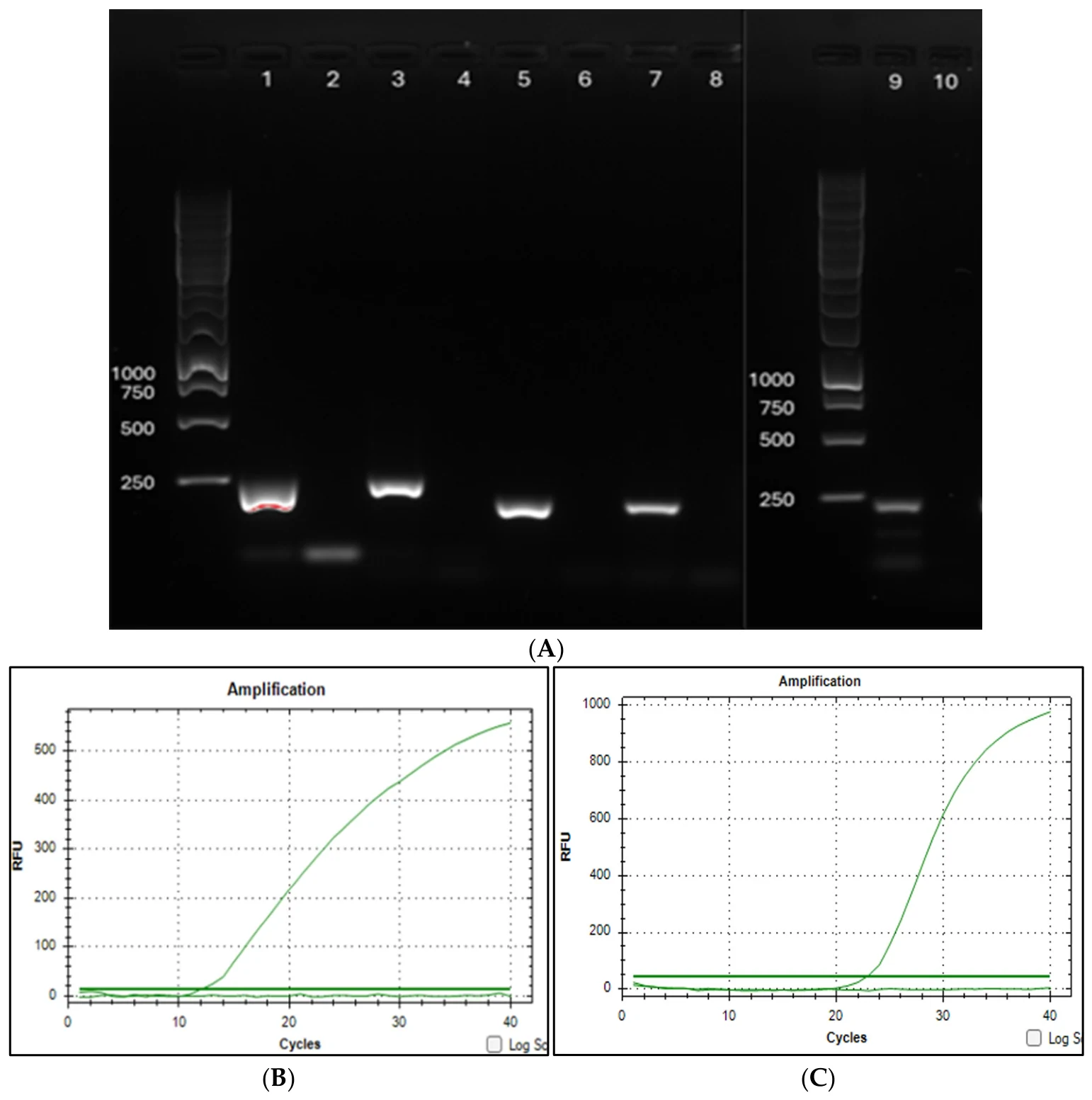
(**A**) Gel electrophoresis (agarose 1.2% in Tris-acetate buffer) analysis of the (RT)-PCR obtained amplicons for lane (1) tomato Leaf Curl (247 bp); lane (3) pepper vein yellow virus (247 bp); lane (5) pepper enamovirus (187 bp); lane (7) cowpea polerovirus 1 (236 nt); and lane (9) Seles weed carlavirus (250 nt). Lines 2, 4, 6, 8, and 10 are the respective no template controls. Panels (**B**,**C**) are the real-time qPCR output for the detection of tomato mottle mosaic (**left**) and potato virus Y (**right**) in tomato sample.

A putative new virus, belonging to the genus *Carlavirus* (elongated particle with a positive-sense ssRNA), and having a BLASTN major hit of the whole genome at 73% nt identity with Ligustrum virus A, was detected and has already been annotated (acc. nr PX870806 [13]. The provisional name ‘Seles weed carlavirus’ was assigned. Further details on the % of mapped reads and BLASTX analysis are reported in Table 1. The graphic representation of the horizontal coverage of the genome is shown in Figure 2A. The specific primers that were selected in this study (reported in Supplementary Table S2) amplified a DNA fragment of the expected size (Figure 3, lane 9).

Apart from this novel putative carlavirus, the presence in the library of begomovirus-derived sequences was hypothesized from the preliminary BLASTN annotation of some contigs. In particular, a few scaffolds matched as major hits (with a size range of 200 nt), including portions of Clerodendron golden mosaic virus (MK626670), and tomato leaf curl Toliara virus (TLC-TV; AM701768), all of them having ca. 75% of average nucleotide identity. When the assembled contigs were analysed with more stringent parameters in BLASTX, a few other hints indicated again the presence in the library of a virus belonging to begomoviruses. Indeed, some contigs matched with Ageratum yellow vein virus, tobacco leaf curl Pusa virus and tomato leaf curl Patna virus. In particular, the homology to these hits mapped in a genomic region spanning around positions 1700–2400 nt. Conversely, a few other contigs blasted to several isolates of chilli leaf curl virus, in a shorter 5′ terminal region (positions around 300 to 500 nt). All these data are summarized in Supplementary Table S3.

Therefore, since the BLAST analysis of contigs assembled from this library returned a significant homology with a begomovirus species, the cDNA of this plant was tested for amplification with the primer set designed on the sequence of Tomato leaf curl Toliara virus (Supplementary Table S2) and thus we obtained a true-size amplicon of about 250 bp (Figure 3, lane 1). Surprisingly, the Sanger sequence of this purified fragment shows a 96.5% nucleotide identity with chilli leaf curl virus isolates (in particular MN494099 and PQ274454) even though the primer set was not intended to be designed in a highly conserved nucleotide sequence region (Supplementary Figure S1). Finally, our evidence about the presence of a begomovirus in the AngD library accounted only for a single genomic DNA. This could belong to an Old World begomovirus, for which no satellites have been found in the analysed sequences.

### 3.2. Viruses in Habanero Pepper (AngH)

In the library derived from the habanero pepper symptomatic plant, the presence of two members of the family *Solemoviridae*, i.e., pepper vein yellows virus (PVYV, belonging to the genus *Polerovirus*) and pepper enamovirus (from the genus *Enamovirus*) were retrieved. These viruses have isodiametric particles and a monopartite, positive ssRNA genome, with seven and four expressed genes, respectively. Both viruses are phloem-limited and efficiently transmitted by aphid species [17]. 

Information about the main statistical values regarding these two viruses is reported in Table 1, and their coverage graphics are shown in Figure 2B,C. For both viruses, the specific primers (Supplementary Table S2) were able to amplify a true-size DNA fragment (Figure 3A, lane 3 and 5) that, when sequenced, demonstrated 98% nucleotide matching with the original viral contigs. This value corresponds also with the percentage identity resulting from the overlapping hits obtained from the BLASTN annotation.

The presence in the library of the cucumovirus cucumber mosaic virus was only assessed by statistical values (Table 1) and not by PCR. The consensus for the three genomic segments (RNA 1, 2 and 3) of this polyphagous virus, which is transmitted by aphids, was reconstructed by mapping the reads against the reference genome (Supplementary Figure S2A). Notably, RNA 2 and 3 of this virus, the latter generally over-represented by the sub-genomic synthesis of the coat protein gene, had a larger prevalence in the coverage (ca. 98%) compared to those of RNA 1 (around 50%). This virus has been recently reported as widespread in African countries on pepper but has not yet been described in Angola on the same crop [18].

Finally, two other virus-derived sequences were highlighted in this library. The first was the Capsicum frutescens alphaendornavirus 1, which mapped with high homology (98.8%) to the reference hit, along with a genomic coverage of 16.3% (Table 1). This virus belongs to a family of single-stranded positive RNA viruses affecting plants and fungi, which are essentially symptomless, do not have a true capsid, and are seed-transmitted [19]. A last viral species that has been identified only by contigs analysis and not investigated by RT-PCR is the Ethiopian tobacco bushy top virus satellite RNA. This small-sized satellite (around 500 nt long), shows a pairwise identity of 94.2% to the major hit, over a genomic coverage of 30% (Table 1; Supplementary Figure S2B). This RNA virus, belonging to the *Umbraviridae* family, requires a helper virus in an association that may enhance the symptom expression [20], and has been described in other crops, rather than tobacco, in Sub-Saharan countries.

### 3.3. Viruses in Bean (AngF)

In the library derived from the symptomatic bean plant, cowpea polerovirus 1 was the only relevant virus identified (Table 1). Recent reports about the occurrence of poleroviruses in legumes suggest this cluster of viruses may be expanding its host range, potentially affecting a broader spectrum of vegetable crops, facilitated by its efficient transmission by aphids [21].

The reads of this virus cover 62% of the genome (Figure 2D) and share 98.5% identity with the major hit in BLASTX. The newly designed primer set (Supplementary Table S2) successfully amplifies a 236 bp DNA fragment (Figure 3A, lane 7), whose sequence corresponds to 99% identity with the original assembled contig.

### 3.4. Viruses in Tomato (AngT)

Tomato is the most cultivated vegetable in Cuanza Sul, with the cultivars IPA6, Roma and Rio Grande being the most widespread. In the single plant sampled in the plot, several viruses were recognized.

Tomato mottle mosaic virus (ToMMV) was the major presence, showing a nucleotide identity of 99% with the strain CpB1, first isolated in South America [22], and already described in Austral Africa, as a major hit in BLASTN. This virus was definitely the most abundant for its number of reads mapping the reference genome among all the libraries (16 million reads attributable to ToMMV out of 58 million total reads in the library) (Supplementary Table S1). The coverage was complete (100% of covered positions; Figure 2E) and thus, the full-length genome sequence was achieved in the contig assembly. To amplify the virus RNA, a RT-qPCR with SYBRGreen detection was applied, with the primers described in [23] targeting a specific 120 bp-amplicon (Figure 3C; Supplementary Table S2).

The coinfection of the potyvirus potato virus Y was also detected in the library analysis. Its RNA genome was covered at 61.7% with a lower number of reads (7985; Supplementary Figure S2C) compared with tobamovirus, mapping anyway to the hit reference genome at over 99% identity. Also for this virus, the primer set, already described in the literature [24] (Supplementary Table S2), was successfully applied for RT-qPCR detection (Figure 3B).

An even lower number of reads (only 114) was associated with the presence of the DNA-genome tomato leaf curl virus. In contrast to the controversial evidence for the presence of a begomovirus in the library AngD, and notwithstanding the reduced amount of reads mapping to the major hit (tomato leaf curl Toliara virus, isolated in Madagascar; acc. nr: NC_038901.1), in the tomato library the genome coverage was higher (67.5%; Table 1). The PCR amplicon (Figure 3A, lane 1), obtained with the primers reported in Supplementary Table S2 and specifically designed on our assembled contig sequence, gave an identity percentage with the BLAST hit of 96.7, comparable with that obtained from the contig alignment (97%).

Finally, potato leafroll virus, which was highly represented in the library (344,951 mapping reads; Table 1), shows consistent coverage (94.4%; Figure 2F) and a 97.7% identity with the reference genome. For this virus, the confirmation through amplification was not carried out.

## 4. Discussion

In our molecular survey, only a few symptomatic plants, potentially affected by viruses, were considered. The usefulness of a HTS generic detection for the identification of a wide array of different viruses within the selected plants was once more demonstrated [25]. The infection of known viruses and the discovery of a new one was achieved by an unbiased tool able to detect either RNA or DNA genomic substrates, as it is the sequencing of rRNA-depleted total RNA.

A limitation of this survey could have been the low number of tested plants, furthermore restricted to symptomatic plants and mainly representing crops. Also, the period of the year that was dedicated to the survey coincided with the beginning of the dry season when the crops were already dried and/or harvested.

Although a larger selection of plants could have been certainly more representative at an epidemiological scale, the aim of the study to search for viruses that could potentially hamper food production was sufficiently accomplished. This small-scale molecular investigation for harmful viruses of some vegetable crops in the visited Angolan areas was consistent with the RE-FARM project activities.

It should be noted that despite considerable variation in the number of viral reads obtained, the data allowed high-confidence identification of the viruses and gave a hint about their proportional titer in the infected plants. Further confirmation for the presence of some of them in the plants, through conventional or real-time PCR, was likewise successful. Moreover, the condition of mixed infection in high-income crops like habanero pepper and tomato is clear-cut and confirms what was already reported [18]. This condition could involve synergistic interaction among different viruses that may have detrimental effects on the crops by increasing symptom expression and could cooperate in a more efficient transmission by the aphid vectors in the field [26].

Among the identified viruses, some should receive major attention. The presence of poleroviruses (in three different plants) pinpoints the relevance and emergence of this group of pathogens, with a stable settlement in sub-tropical areas and a wide diffusion due to efficient aphid transmission [18]. Fiallo-Olivè et al. (2018) [17] stated that the yellowing disease of pepper emerging worldwide is caused by a complex of different poleroviruses, and differentiation of species would arise from recombination events. In symptomatic pepper, a recent investigation through HTS in Hungary discovered a quite similar situation of mixed infection which does not involve poleroviruses [27], but shows persistent viruses like an endornavirus in the viral community. In contrast, Lotos et al., (2017) found in infected pepper in Greece only the presence of a new species of polerovirus associated with pepper yellowing disease [28]. A recurrent occurrence of PVYV was noticed in Benin in mixed infections [29], and its prevalence indicated that PeVYV is a major constraint to pepper production in the country.

On the other hand, the spread of the begomoviruses, phloem-limited viruses efficiently transmitted essentially by the whitefly *Bemisia tabaci*, should be quite concerning for the persistence of these viruses, the high potential of recombination and the rise of new aggressive strains or species [30,31]. A signal in this direction is the evidence for the presence of a begomovirus in the wild weed (AngD library). The quite limited number of reads did not allow a precise classification of the agent that was tentatively identified as chilli leaf curl virus based on a very short genomic region amplified. It could be perhaps a recombinant sequence or a clue for a coinfection by two different begomoviruses, since two different portions of the genome were addressed by homology. Full-length genome sequencing, phylogenetic analysis of these sources and the possible association of satellite(s) are therefore future desirable studies on the same plants in the area.

Emergence and outbreaks of begomoviruses have also been recently confirmed to be associated with seed transmission [32], as several combinations of plant and virus genotypes could favor a passage from an infected plant to seedlings, supposedly via seed coat contamination.

Concerning the origin of the retrieved viral sources, few sources can be hypothesized: the only starting point could be the genetic affinity of the major matching hits with the local isolates. It is conceivable that most of the isolates moved through the closest African countries, most likely due to the uncontrolled cross-country seed trade. Conversely, the widespread ToMMV isolate detected on tomato, as already reported, has a Brazilian origin. 

Two other issues facing the survey are of particular concern. One is the farmer seed self-propagation, which is generally a major source of carry-over for viral and systemic pathogens. Other principal risk factors are the strict rotations of the same crops on the fields and the absence of cheap and controlled nursery plantlet supplies. The role of supporting and regulating rural agencies can be strongly envisaged in this context.

A final concern comes from the hidden role as viral reservoirs played by wild weeds [33]. The discovery of a previously unknown, aphid-transmitted carlavirus in a symptomatic weed on the border of cultivated fields raises concern about any potential spill-over into the crops or plants of botanical affinity [13]. The expansion of agricultural land toward the semi-arid savannah or forest wildlands has been indicated as a connection borderline where wild weed and crops may reciprocally exchange transmissible pathogens [7]. Indeed, despite our relatively small survey experience, our findings suggest that monitoring of the crops by at least visual inspection and then, molecular diagnostic tools, could contribute to the epidemiological knowledge of plant pathogens and their effective management and containment [34]. The implementation of sanitary certification for seeds and plantlets production, to reduce the primary inoculum source in the fields, as well as the introduction of virus-resistant cultivars, will be in the future a factor of importance for food yield and quality.

## Figures and Tables

**Table 1:** Summary of the viruses identified by high-throughput sequencing in the four libraries. For each detected virus, genus and species (vernacular name), reference accession number, number of mapped reads, pairwise nucleotide identity with the closest hit, genome coverage, and average BLASTX e-value of the assembled contigs are reported. Average BLASTX e-values are expressed in scientific notation. Lower e-values indicate higher similarity to the corresponding viral protein sequences. The accession numbers are generally those of the reference genomes used for the reads mapping, while the geographic origin in brackets is related to the acc. nr. of the major hit matching the longest contig of each virus.

Library Name	Virus Genus	Virus Species Identified	Acc. Nr. (Geographic Origin)	Nr. Mapped Reads	Pairwise Identity With Closest Hit (nt) %	Genome Coverage %	Average E-Value BLASTX Contigs
*AngD*	*Carlavirus*	Seles weed carlavirus	PX870806.2 (Angola)	13,694	98.9	99.9	3.80 × 10^−304^
*AngF*	*Polerovirus*	Cowpea polerovirus 1	NC_034246.1 (Burkina Faso-KY3648469)	15,478	98.5	62.5	8.92 × 10^−299^
*AngH*	*Alphaendornavirus*	Capsicum frutescens endornavirus 1	MN175322.1 (Ecuador)	3969	98.8	16.3	2.35 × 10^−302^
*AngH*	*Polerovirus*	Pepper vein yellows virus genomic RNA	NC_015050.1 (India–ON96154)	487,946	98.2	93.5	3.44 × 10^−184^
*AngH*	*Umbravirus*	Ethiopian tobacco bushy top virus satellite RNA	NC_024807.1 (Ethiopia)	326	94.2	34.5	1.91 × 10^−134^
*AngH*	*Enamovirus*	Pepper enamovirus	NC_037052.1 (Rwanda–MG470803)	199,851	98.5	97.0	1.54 × 10^−135^
*AngH*	*Cucumovirus*	Cucumber mosaic virus RNA 1	NC_002034.1 (Rwanda–MG470798)	991,537	97.3	52.0	3.42 × 10^−25^
*AngH*		Cucumber mosaic virus RNA 2	NC_002035.1 (Rwanda)	1,067,662	99.2	97.3	3.42 × 10^−25^
*AngH*		Cucumber mosaic virus RNA 3	NC_001440.1 (Rwanda)	2,321,117	99.1	98.6	3.42 × 10^−25^
*AngT*	*Potyvirus*	Potato virus Y	NC_001616.1 (Zimbabwe–MG602675)	7985	99.2	61.7	4.43 × 10^−62^
*AngT*	*Tobamovirus*	Tomato mottle mosaic virus	NC_022230.1 (Brazil–MH128145)	16,264,386	99.0	100	2.71 × 10^−101^
*AngT*	*Begomovirus*	Tomato leaf curl Toliara virus	NC_038901.1 (Madagascar–AM701768)	114	98.4	67.5	1.35 × 10^−124^
*AngT*	*Polerovirus*	Potato leafroll virus	NC_076505 (Bangladesh–PX317653)	344,951	97.7	94.4	6.45 × 10^−70^

## Data Availability

This metagenome project has been deposited in the GenBank repository. Ang_D library (BioProject: PRJNA1399677; BioSample: SAMN54503719; SRA: SRR36723783); Ang_H library (BioProject: PRJNA1470426; BioSample: SAMN60413132; SRA: SRR38839858); Ang_F library (BioProject: PRJNA1470426; BioSample: SAMN60413131; SRA: SRR38839859); Ang_T library (BioProject: PRJNA1470426; BioSample: SAMN60413133; SRA: SRR38839860).

## Supplementary Materials

The following supporting information can be downloaded at https://www.mdpi.com/article/10.3390/v18080822/s1, Figure S1: Multiple sequence alignment of the amplified genomic region of a putative begomovirus from AngD sample vs. the sequences of matching hits; Figure S2: Coverage maps of HTS reads aligned to different reference genomes; Table S1: Summary of the sequencing statistics for the four high-throughput sequencing libraries; Table S2: List of primers used in this study; Table S3: Summary of begomovirus-related contigs detected in the AngD library.
