# Supplementary material for: Plant Viral Metagenomic Analysis from a Preliminary Field Survey in Angola Reveals Complex Mixed Infections in Vegetable Crops
Source: Viruses. 2026 Jul 26;18(8):822. doi: 10.3390/v18080822 (PMC13517959; doi:10.3390/v18080822)
Supplement: Supplementary file 1 [file viruses-18-00822-s001.zip › viruses-4439608-supplementary.pdf]

viruses

MDPI

Communication

Plant viral metagenomic analysis from a preliminary field survey in Angola reveals complex mixed infections in vegetable crops

Serafina Serena Amoia [1], Annalisa Giampetruzzi [1], Fernando Francisco de Sousa Neto [2], Luisa Flora António [2], Adérito Tomás Pais da Cunha [2,3] and Angelantonio Minafra [1,*]

[1] Institute for Sustainable Plant Protection–CNR, 70126 Bari, Italy; serafinaserena.amoia@cnr.it (S.S.A.); annalisa.giampetruzzi@cnr.it (A.G.)
[2] Instituto Superior Politécnico do Cuanza Sul, Sumbe P.O. Box 82, Angola; fernando.neto@ispcs.ao (F.F.d.S.N.); flora.antonio@ispcs.ao (L.F.A.); aderito.cunha@ciencia.ao (A.T.P.d.C.)
[3] Centro Nacional de Investigação Cientifica, Luanda P.O. Box 34, Angola
* Correspondence: angelantonio.minafra@cnr.it

Supplementary Table S1. Summary of the sequencing statistics for the four high-throughput sequencing libraries

Library Name	Total reads	Total reads after adapter trimming	Total bases	GC content %
AngD	58,196,570	58,129,988	8,787,682,070	46,4
AngF	44,763,126	44,633,978	6,759,232,026	43,5
AngH	55,713,104	55,581,226	8,412,678,704	44,2
AngT	58,179,164	58,036,456	8,785,053,764	43,5

Supplementary Table S2. List of primers used in this study.

Virus name	Sequence (5′-3′)	Amplicon size	Reference
Seles weed carlavirus	D-carla_for<br>TAAAGTAACTTTCCTGAT GGCTG<br>D-carla_rev<br>CCAATCCCTGCATGAGCGTTCAC	250	This study
Cowpea polerovirus 1	F_COP1_FOR<br>GTGGTTAAGAAGGAATGGAGG<br>F_COP1_REV<br>GACGGCCCGAACGTGATTGCTC	236	This study
Pepper enamovirus	H_Pen_for<br>AAATCTGTGAAGTCGGGAGCAT<br>H_Pen_rev<br>CCCTAGGCCTTTCATTCCTGTT	187	This study
Pepper vein yellows virus	H_PEVY_for<br>ACTGATTCGAGGGCATCATCTC<br>H_PEVY_rev<br>TCCGCAATAAACTCACACTGGA	247	This study
Tomato mottle mosaic virus	ToMMV-rtfor<br>TTGTCATCAGCATGGGCCGACC<br>ToMMV-rtrev<br>ACACCTCGCTGAACTGCTGTTG	116	Aceto et al., 2024
Tomato leaf curl Toliara virus	T_TLC_for<br>GTACATCTTGGGCTTTCGGTAC<br>T_TLC_rev<br>GAGAAAGAGTAGAGCCAGGTA	247	This study
Potato virus Y	PVY_for<br>AGGGATGGTTGATCGACTGGGA<br>PVY_rev<br>CCACTGCCATTCATGAGCTGT	116	Prigigallo et al., 2019

Supplementary Table S3. Summary of begomovirus-related contigs detected in the AngD library. The closest reference sequence (as accession number and geographic provenience of the isolate), number of mapped reads, pairwise nucleotide identity, percentage of genome coverage, and average BLASTX e-values of the assembled contigs are reported.

Library name	Virus genus	Virus species identified	Acc. Nr. (Geographic origin)	Nr. mapped reads	Pairwise identity with closest hit (nt) %	Genome coverage %	Average e-value BLASTX contigs
AngD	Begomovirus	Tomato leaf curl Toliara virus	AM701768.1 (Madagascar)	1,471	99.0	9.4	1.17^{e-17}
AngD	Begomovirus	Tobacco leaf curl Pusa virus DNA-A	NC_014596.1 (India)	319	99.4	17.1	1.74^{e-53}
AngD	Begomovirus	Ageratum yellow vein virus	JN809816.1 (Thailand)	1,380	95.8	20.7	2.47^{e-65}
AngD	Begomovirus	Clerodendron golden mosaic virus	MK626670.1 (China)	138	98.6	14.4	5.09^{e-44}
AngD	Begomovirus	Tomato leaf curl Patna virus	KM383745.1 (Bangladesh)	3152	96.5	19.3	2.34^{e-53}
AngD	Begomovirus	Chilli leaf curl India virus isolate SSPB 17	PQ274454.1 (India)	264	97.6	6.9	1.73^{e-37}
AngD	Begomovirus	Chilli leaf curl India virus isolate Lahore	MN494099.1 (India)	459	97.8	10.6	3.0^{e-29}

```
038                                     GAGAAAGAGTTACGAGCCAGGTACCCTTGGTTG     60
039        -----------------------------------------------------------C       1
MN49       ------------------------------------------GTAGAAAATACGTATTCCCCAGATACTCTGGGCTA    18
PQ27       -------------------------------------------GTAGAAAATACGTATTCCCAGATACTCTGGGCTA    17

038        GGAATTAATTCGTGATTTATTGCTAGTTATACGTGCGAAGAACTATGTCGAAGCGACCAG      120
039        GGATGAGAGGTTACGTAAGAAAACAAGTTGCTCTGCGAAGAAATATGTCGAAGCGACCAG      61
MN49       CGATTTAATTAGGGATTTAATTTCAGTTATCAGGGCTAGGAATTATGTCGAAGCGACCAG      78
PQ27       CGATTTAATTAGGGATTTGATTTCAGTTATTCGGGCTAGAAATTATGTCGAAGCGACCAG      77
            **    *       *        *  *      ** *  ** *****************

038        CAGATATAGTCATTTCCACGCCCGTCTCGAAGGTACGACGTCGTCTGAACTTCGACAGCC      180
039        CAGATATAGTCATTTCCACGCCCGTCTCGAAGGTACGACGTCGTCTGAACTTCGACAGCC      121
MN49       CAGATATAATCATTTCCACGCCCGCCTCGAAGGTACGCCGTCGTCTGAACTTCGACAGCC      138
PQ27       CAGATATAATCATTTCCACGCCCGGCTCGAAGGTACGTCGTCGTCTGAACTTCGACAGCC      137
           ******** *************** ************ **********************

038        CATATGCCAGCCGTGTACGTGCCCCCACTGTCCTCGTCACAAACAGAAAGAGGGACATAG      240
039        CATATGCCAGCCGTGTACGTGCCCCCACTGTCCTCGTCACAAACAGAAAGAGGGCTTGGG      181
MN49       CATATGCCAGCCGTGCTGCTGCCCCCATTGTCCGCGTCACAAAGGCAA---GGGCATGGA      195
PQ27       CATATGCCAGCCGTGCTGCTGCCCCCATTGTCCGCGTCACAAAGGCAA---GAGCATGGG      194
           ***************    ******** ***** *********   **   * *

038        AAATGAATT---------------------------------------------------      249
039        CCAACAGGCCCATGTACCGAAAGCCCAAGATGTAC-------------------------      241
MN49       CCAACAGGCCCATGAACCGAAAGCCCAGAATGTACA------------------------      231
PQ27       CGAACAGGCCCATGAACCGAAAGCCCAGGATGTACA------------------------      230
```

Supplementary Figure S1. Multiple sequence alignment obtained with Clustal Omega tool of the amplified genomic region from AngD sample using T_TLC for/rev primers, sequenced in both directions (038 and 039), and the closest matching begomovirus isolates [(MN49- MN494099.1 Chilli leaf curl India virus isolate Lahore) and (PQ27- PQ274454.1 Chilli leaf curl India virus isolate SSPB 17 segment DNA-A)]. Conserved nucleotides are indicated by asterisks, while nucleotide substitutions and indels are highlighted. Colored regions indicate the primer binding sites used for PCR amplification.

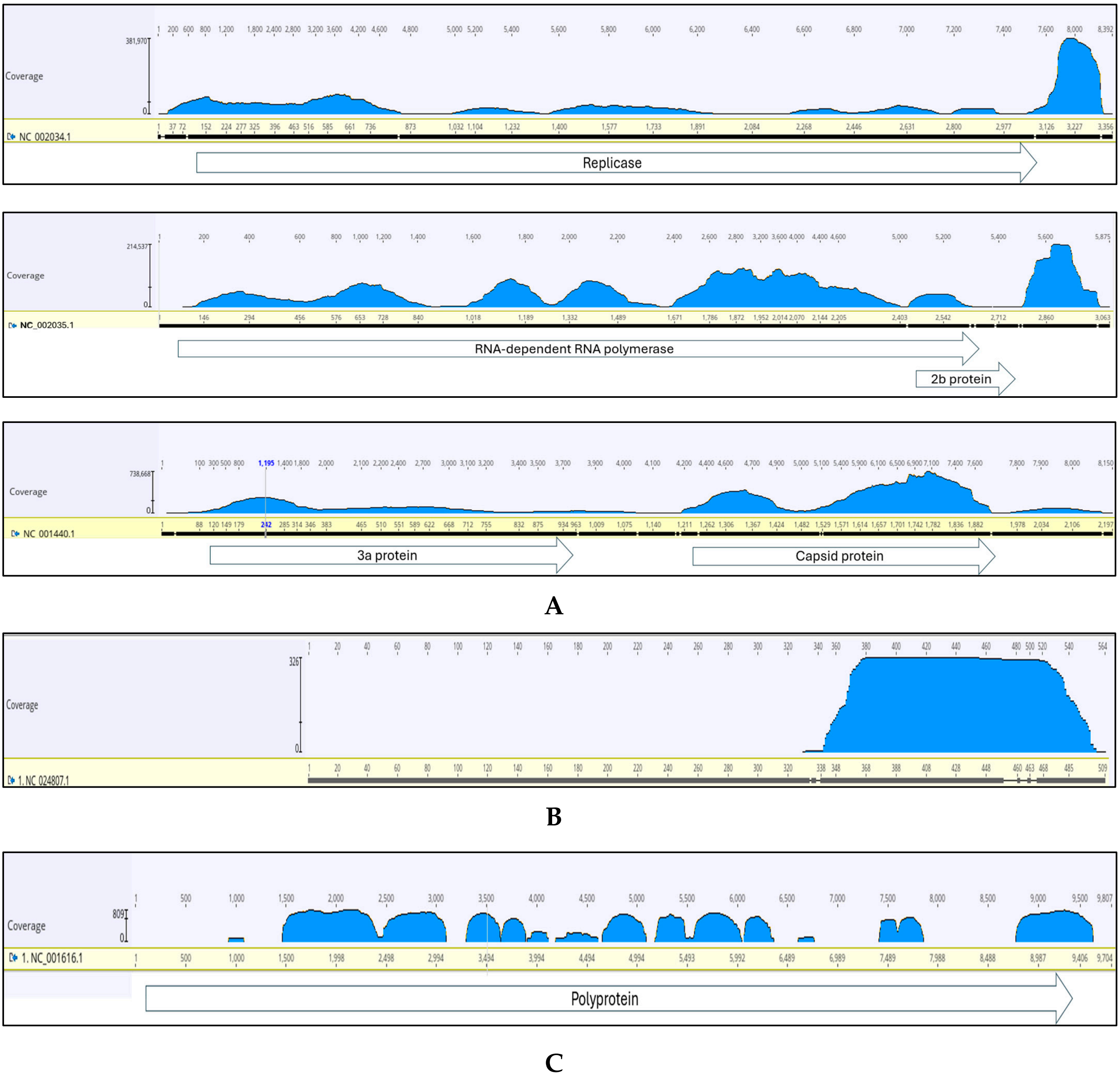

Supplementary Figure S2. Coverage maps of HTS reads aligned to the complete genomes of A) the three genomic segments (RNA 1, 2 and 3) of cucumber mosaic virus; B) Ethiopian tobacco bushy top virus satellite RNA; C) potato virus Y. The blue histograms represent the coverage profile along the reference viral genomes, whereas arrows indicate the positions of the annotated genes and ORFs.
